# Developing and sustaining a community advisory committee to support, inform, and translate biomedical research

**DOI:** 10.1017/cts.2022.473

**Published:** 2022-10-11

**Authors:** Joseph A. Skelton, Stephanie S. Daniel, Hazel Tapp, Keena R. Moore, Lilli Mann-Jackson, Jorge Alonzo, Isaiah Randall, Victor Isler, Claudia Barrett, Diamond Badger, Elizabeth Lees, Scott D. Rhodes

**Affiliations:** 1 Department of Pediatrics, Wake Forest University School of Medicine, Winston-Salem, NC, USA; 2 Clinical and Translational Science Institute, Program in Community-Engaged Research, Wake Forest University School of Medicine, Winston-Salem, NC 27157, USA; 3 Department of Family and Community Medicine, Wake Forest University School of Medicine, Winston-Salem, NC, USA; 4 Department of Family Medicine, Atrium Health, Charlotte, NC, USA; 5 Department of Social Sciences and Health Policy, Wake Forest University School of Medicine, Winston-Salem, NC, USA; 6 Office of County Manager, Guilford County, Greensboro, NC, USA; 7 Imprints Cares, Winston-Salem, NC, USA; 8 Forsyth Futures, Winston-Salem, NC, USA

**Keywords:** Community engagement, translational research, advisory board, methods

## Introduction

Community-engaged research (CEnR) features teams of diverse partners and constituencies,^1^ including community members, patients, organization representatives, and academic partners, with a wide range of expertise in and perspectives on the research process. Members from communities become engaged as partners or team members, rather than “targets” of research. CEnR emphasizes values such as trust and relationship building, open communication, teamwork, collaboration, and cooperation [1–8].

By including diverse voices in CEnR, research questions may emerge that are more relevant to community and population health. In addition, study designs are more likely to be feasible and have support from communities [5,9,10]. CEnR also may facilitate greater uptake of study findings by partners and constituencies, including community member advocates, organizations, and practitioners.

Community engagement often is viewed as a continuum that begins with outreach (less engaged) and may move through consultation, involvement, and collaboration to shared leadership (most engaged) [11,12]. There is tremendous variation in how CEnR is defined, implemented, and evaluated [1,2,6,7,13]. Nonetheless, CEnR includes critical elements such as:

- collaboration with diverse groups and individuals, including community members who may be affiliated by geographic proximity, special interests, health conditions, or other categories of shared identity;
- a focus on addressing the needs and priorities and harnessing the assets that affect the health and well-being of communities; and
- partnerships and coalitions that foster translation of findings into programs, policies, and practices [3,13].

A fundamental way to facilitate CEnR is by forming community advisory committees (CACs) that include representatives from all relevant parties in a meaningful way. CAC members may share the perspectives of agencies and organizations or their own perspectives based on personal and lived family experiences with research, health care and health needs, disease prevention and management, and community interactions and involvement. As such, CACs can guide individual studies or even entire research programs, thereby increasing researchers’ understanding of community needs and priorities. CACs can allow community partners to respond to gaps and address barriers by generating their own research ideas. CACs can also facilitate community-academic research partnerships and collaborations; ensure better cultural congruence, meaningfulness, and effectiveness of research phases and activities; and contribute to the dissemination of research findings.

## Learning Health Systems

Academic health centers are emphasizing their role as LHSs, wherein they use internal and external evidence to improve the practice and safety of medical care [14,15]. But within this setting, developing and harnessing the wide range of expertise and perspectives of members of CACs may be challenging. For example, the community may have lingering distrust of medical research or of the LHS itself; clinics or hospitals in urban areas or nearby communities may have closed or relocated to suburban or wealthier areas; or research conducted may not be of interest to communities or reflect their needs or priorities. Even if a CAC is developed, the expertise and perspectives of members may not be adequately elicited, recognized, or effectively used. More guidance is needed for academic researchers and their community partners to successfully build, engage, and sustain a CAC to improve community and population health and well-being, aid in disease prevention and progression, increase health equity, and reduce health disparities.

In this paper, we provide guidance on developing and main-taining an effective CAC designed to ensure community engagement. We describe the experience of the Program in Community-Engaged Research (PCER), a core program of the Clinical and Translational Science Institute (CTSI) at Wake Forest University School of Medicine (WFUSM).

## Developing and Maintaining a CAC

Rhodes et al developed a multistep process to guide CEnR processes, based on 24 CEnR projects over a 17-year period [7]. This process begins with networking and partnership expansion, continues with building trust and rapport, and extends through the entire research process, concluding with dissemination of findings and translation. We adapted these steps to describe the development and maintenance of the CAC that guides the Wake Forest CTSI.

### Networking and Partnership Establishment and Expansion

Our CAC was begun in 2010 by investigators with active CEnR collaborations. It was built on a history of mutually beneficial partnerships and research to reduce pesticide exposure among Latine^2^ farmworkers; improve obesity treatment among children [16–18]; and prevent and treat infectious diseases (e.g., HIV and sexually transmitted infections) among racial/ethnic, sexual, and gender minorities and those living in rural communities [7,9,19,20]. These existing relationships were harnessed to establish a working group of community members, organization representatives, and academic researchers who met monthly to establish rules and guidelines for community-academic collaboration. The initial group then identified other organizations that could be added to the CAC and talked through their rationale. For example, the local YMCA was identified as a potential member of the CAC given their focus on community well-being, including physical activity, obesity prevention, mentorship of youth, and literacy, with a history of programming and partnerships with WFUSM researchers.

Representatives at each organization were identified and personally invited to participate by community members already engaged in the CAC. Organizations received invitations to participate in one CAC meeting but were not required to become a member. Community members were also encouraged to bring colleagues as guests. For example, the executive director of a local food bank and anti-hunger organization was invited to attend a CAC meeting by a current CAC member (and friend); after this initial meeting, the executive director became a member of the CAC. One participant described the friendly atmosphere as, “… like being an invited guest to a Rotary club meeting; you aren't a member but were made to feel welcome as a guest.” After the CAC was established, a snowball methodology was used to involve potential new members. Current CAC organization members are listed in Table 1.

**Table 1.** Wake Forest University School of Medicine (WFUSM) community advisory committee (CAC) membership

Agency	Focus area
American Cancer Society	Cancer research, education, prevention, and support
Authoring Action	Arts, youth, social change
Boys and Girls Club	Youth development
Center for Design and Innovation	Arts, science, education, community
Community Advocate	Faith, education
Comprehensive Cancer Center	Cancer research, education, prevention, patient navigation
FaithHealthNC	Faith-based, access to care, community resources
Forsyth County Department of Public Health	Preventive health, environmental health, refugee support, family planning
Forsyth County Department of Social Services	Adult and child protective services, guardianship, adoption, foster care, counseling, economic support
Forsyth County Government	Policy
Forsyth Futures, Inc.	County action-oriented data analysis and reporting
H.O.P.E of Winston-Salem	Food insecurity
Habitat for Humanity of Forsyth County	Housing insecurity
Imprints Cares	Early childhood education
Inmar	Technology and data science
Insight Human Services	Substance abuse, mental health
Kaleideum	Youth, STEM
Kate B. Reynolds Charitable Trust	Equitable access to health and health outcomes, Funding
Lead Girls of NC Inc.	Youth education and mental health
Map Forsyth	Resource mapping, data
Mental Health Association of Forsyth County	Mental health
Mi Casa Sevicios Hispano-Latinos	Immigration, advocacy
North Carolina Cooperative Extension	Agriculture, youth, family
Northwest Area Health Education Center	Continuing medical education and training
Novant Health	Medical center
Piedmont Environmental Alliance	Environmental health
Piedmont Triad Regional Council	Aging, criminal justice, economic and community development, regional planning, workforce development
Salem College	Higher education
Second Harvest Food Bank	Food insecurity
Senior Services, Inc.	Aging
Smart Start of Forsyth County, Inc.	Early childhood education
The Partnership for Prosperity	Poverty, advocacy
Trellis Supportive Care	Palliative care, family support
United Way of Forsyth County	homelessness, third grade reading proficiency, high school graduation rates, poverty, funding
University of North Carolina School of the Arts	Higher education
Wake Forest University	Higher education
Wake Forest University School of Medicine	Research, patient-care
Winston Salem Foundation	Funding, disparities: education, economic, residential
Winston Salem State University	Higher education
Winston Salem/Forsyth County Schools	K-12 education
Winston Salem Police Department	Public safety
YMCA of Northwest NC	Faith, healthy physical and mental health behaviors

### Structure and Organizational Framework for a CAC

While coordination and communication are facilitated by WFUSM staff and faculty, CAC leadership (chair, chair-elect, and member-at-large) is entirely comprised of community members. Each position has a 2-year term to ensure continuity. Leaders typically volunteer or are nominated by CAC members, with approval by vote at a regularly scheduled CAC meeting. Leaders are responsible for establishing meeting agenda topics, leading CAC meetings, and gathering input from members.

CAC meetings are held quarterly, and last 60–90 minutes. Until the onset of the COVID-19 pandemic, they were always held in a community location and refreshments were served. Since the onset of the pandemic, meetings have been held virtually. Although some conveners of CACs pay community members to participate, we do not. We made this decision because our CAC members represent organizations, and in most cases, attendance may be part of their jobs. However, individual community-academic research teams may engage community members as part of specific intramurally and extramurally funded projects. In these cases, community members may be compensated for such work.

Our CAC schedules meeting dates, times, and locations a year in advance, with regular reminders sent to members. The general structure of the meetings and a sample agenda are presented in Table 2.

**Table 2.** Community stakeholder advisory committee meeting agenda and description

Agenda Item	Details	Explanation
Welcome and Introductions	Each partner attendee introduces themselves and the organizations they represent	To build relationships among partners and the potential for inter-organization collaboration
Funding	Updates may include programmatic CTSI funding awarded to community partners, extramural CEnR funding received, etc.	
Guest speakers or structured discussion	Often will include a brief presentation from an academic partner and an organization representative about some topic of mutual importance (e.g., physical activity among older adults, Medicaid transformation) or small group discussions to establish CSAB priorities or guide the PCER	These presentations and discussions build community-academic partnerships, increase awareness of community needs, priorities, and assets; and guide research and programmatic next steps
Dissemination	Highlights of dissemination activities, including publications, policy briefs, and infographics	
Announcements	Members provide brief organization updates	

The CAC leaders meet monthly to plan and discuss quarterly meeting agendas, identify speakers and presenters, address challenges, and celebrate successes. The COVID-19 pandemic required changes to PCER activities, for example, CAC leadership helped make decisions such as pausing the Community Tours previously held twice yearly [21].

The CAC leadership serves the PCER in a formal capacity by reviewing and scoring pilot grant proposals and contributing to grant applications for external funding; helping to prepare manuscripts; disseminating findings, outcomes, and products; and generating ideas for potential projects. The CAC leadership also convenes a Core Working Group that includes community members, PCER leaders, and awardees of intramural PCER grants. While the CAC agenda is organized around community needs, priorities, and assets, the Core Working Group is tasked with moving specific projects and initiatives forward. This group has a close-knit and informal structure, with a culture of mutual respect and shared decision-making. Our CAC leadership limits the extent of academic faculty and PCER staff involvement in meetings and activities to avoid shifts in dynamics and discussions, which could erode trust and feelings of community control and involvement.

The CAC membership structure includes how long members serve, which can vary and can be devoted to a specific project. Once that project is completed, members can rotate off the CAC if desired. Guidelines governing regular participation may help or hinder a CAC. Provisions for members to participate as much as they can or wish can augment a welcoming culture; however, other policies may be needed if members are paid for participation. In addition, if CACs are too small, they may not sufficiently represent the diversity of community voices; if they are too large, members may not be able to participate as desired. Thus, membership guidelines should be designed to meet the needs of the community and institution. WFUSM’s CAC membership guidelines are deliberately kept simple, as members are not paid, and want them to feel welcome and participate as much as they are comfortable. Individuals are asked to be members, representing their organization, but without criteria for participation in meetings or activities. They are asked annually if they wish to continue being a member and receiving communications and are removed from membership by their request, with an invitation to re-join in the future should they desire.

### Fostering an Engaged CAC

Besides the quarterly meetings of the full CAC, ongoing dialog between members and PCER staff reinforces trust and nurtures relationships, demonstrates responsiveness, and ensures progress. When topics of interest are identified, we have learned that having a community partner and academic researcher each provide a short presentation can be informative. For example, a representative of Senior Services, Inc. gave a 15-minute interactive presentation on needs of local seniors; this presentation was followed by another brief presentation by a WFUSM gerontology researcher on their latest NIH-funded study. This format can increase awareness of an issue or population, identify overlapping interests and differences in perspectives, share supportive data and resources, and help determine next steps. Community members’ requests for future presentations on topics are planned and organized as available.

Maintaining a CAC requires consistent and clear communication with members. Email messaging is the most common and easiest method, although some CAC members may prefer telephone calls or text messages. It is also key to reach out to community organizations with staffing and leadership turnover, to identify new agency representatives and CAC members.

Below we provide two examples of ongoing projects that grew from our CAC.

#### Violence as a Health Disparity

In response to the Sandy Hook school shooting in 2012, the CAC developed a work group to address and better understand violence as a health disparity. This group has collaboratively given community presentations and forums to discuss data related to gun violence and strategies for addressing this issue. They received a grant from the state of North Carolina to support the NC Injury Prevention Academy, which supported further team development among community and academic partners and helped them learn about evidence-based practices for addressing gun violence as a health disparity. The work group also created a photovoice project with a partner organization to document minority youth perspectives about violence and its causes, consequences, and potential solutions [22]. The work group contributed to evidence-based information on a website providing resources for parents’ concerns about the mental health needs of their children – a need identified by one of our community partners. They also organized a forum for discussing recent gun violence events in the community. Partners continue to share leadership in the direction and focus of research activities.

#### Science Outreach

In response to the low numbers of underrepresented minority learners pursuing science, technology, engineering, and mathematic (STEM) careers, CAC members developed the Science Outreach Working Group. This group seeks to enhance elementary, middle, and high school teacher capacity and further learning and engagement in STEM among students, especially from historically marginalized communities. The group meets bi-monthly with representatives from local universities, the local school district, and Wake Forest researchers. To date, hundreds of K-12 learners have participated in tours of Wake Forest research facilities, STEM educational open houses, career expos, summer enrichment camps, and in-class co-facilitated instruction with Wake Forest students in partnership with K-5 teachers.

## Facilitating Research through Our CAC

Additional components of the CAC that further contribute to collaboration in CEnR are described below.

***Community Research Associates (CRAs)*** are community members employed part-time (typically 20 hours a week) by the CTSI to work with community organizations to facilitate and support CEnR. CRAs undergo research training and become certified in human subjects protection. They spend time with leaders in community organizations who may or may not be involved in the CAC. They listen to community concerns and engage with WFUSM researchers, serving as a link between WFUSM and local communities. CRAs also may assist in research activities, such as data collection, consenting/assenting research participants, and disseminating study findings within communities. Typically, they spend 2 years in this role to allow for different individuals or organizations to participate but are able to extend the time if desired.

A successful project led by a CRA was a “citizen science” project that recruited over 100 volunteers to be trained and subsequently observed community use of five local greenways. Key findings were published [23] and applied in subsequent research (e.g., a grant application) and practice (e.g., through better outreach to communities with greater barriers to greenway use).

***Community-Engaged Research Fellowships*** provide partial salary support for a representative from a community organization and a WFUSM faculty member, to develop a 12-month project prioritized by a community and of mutual interest. Each partner typically spends around 8–12 hours a week on this shared project, and experiences and project findings are disseminated within community and academic settings. The fellowship can be extended for second year depending on need and ongoing progress. Community and faculty linkages are often facilitated through the CAC, and recipients of the fellowship participate in CAC meetings and activities.

***Community-Engaged Cooperative Agreements*** provide $30,000 to support a CEnR research partnership with at least one representative from a community organization and one academic partner who are co-Principal Investigators. An example of a recent cooperative agreement involved a partnership between a representative of a community organization, Forsyth Futures, and an academic investigator to assess unmet health and social needs of young women entering adulthood in an underserved section of Winston-Salem, North Carolina [24].

***Community Engagement Boost Awards*** provide $5,000 to supplement translational work of community organizations and partners. These awards, given quarterly, can support implementation of evidence-based interventions, fund evaluations, prepare pilot data for an external grant application, or advance changes that promote health. Examples of successful boost awards are a project to investigate vaping practices in local high schools, led by administrators at the school partnered with a researcher; and another to engage grade school “Girls as Citizen Scientists” to identify solutions to community environmental health priorities. Both of these supported projects went on to receive subsequent external funding.

***Research Awards*** go to community-academic partnerships working to meet an emergent community priority related to a local acute event or crisis. This is a new funding mechanism, and so far only one award has been funded, to capture the impact of an industrial fire in a densely-populated, lower-resourced neighborhood in Winston-Salem, NC (https://response.epa.gov/site/site_profile.aspx?site_id=15489).

Pilot funding announcements are shared through the CAC, encouraging community organizations to work with faculty research partners to apply for funding. The CAC also assists organizations to identify an academic partner if needed. A core group of CAC leaders reviews applications, serves as equal voting members regarding funding decisions, and provides assistance and guidance during projects.

### Critical Successes of Our CAC

Developing a CAC and harnessing the insights of its members can be challenging. Here, we share some successes.

*HOPE (Help Our People Eat) of Winston-Salem* works with community volunteers to prepare and deliver nutritious weekend meals to children in Forsyth County, NC, who are at risk of hunger. The Executive Director of HOPE began attending CAC meetings after being invited by a local leader. He was then paired with two faculty in the WFUSM pediatrics department who wished to better understand and help address food insecurity in their clinics and within the community. The connection and idea led to a Community-Engaged Research Cooperative Agreement project and a Community-Engaged Research Fellowship. The team then received funding from a foundation for the project and ongoing research collaborations [25–27]. The linkages have led to new clinical projects, educational opportunities for medical students, and subsequent grant submissions, including a funded NIH R01 award to explore the cardiometabolic consequences of food insecurity among persons with HIV, and a funded foundation grant to support a cooperative grocery located within a food desert.

*Authoring Action* is a community organization that uses the arts and creative writing for self-expression and social change among local youth. The Director of Authoring Action initially participated in a Community-Engaged Research Fellowship to develop an evaluation plan for their organization. A WFUSM faculty member helped to provide training in developing an evaluation plan. This work resulted in a shared research project and publication regarding youth perspectives of violence as a health disparity within our community [25]. Youth participating in Authoring Action activities made presentations to the CAC and in a community venue. Authoring Action has now received a foundation grant to further their work.

*BeInvolved* is a bilingual web-based database created by the WFUSM CTSI, which aims to bridge study recruitment needs with community understanding of and access to research. *BeInvolved* administrators asked the CAC for feedback to make the site more useful for community members. The CAC provided suggestions on images, format, language, tag lines, and site navigation. This feedback was incorporated and has increased website traffic and helped to bolster enrollment in research studies among underrepresented groups. The CAC now facilitates ongoing community review of *BeInvolved* updates and revisions. This work was accepted as an abstract at a recent American Public Health Association Annual Meeting [28].

Finally, the *Center for Addiction Research* at WFUSM had set goals and objectives for community outreach and dissemination. The CAC invited the Center Director to join a discussion on disseminating results of substance use research into communities. The Director and community members later reported that they found the discussion informative, destigmatizing, and exciting. Some CAC members personally affected by addiction through family and/or friends reported being better able to advocate for those affected by addiction.

### Lessons Learned

Four critical lessons have been learned in our experiences with the CAC:

- *Keep engagement ongoing*. Immediate results, partnerships, or outcomes may not emerge. Thus, it can be useful to design metrics to capture new and sustained relationships and partnerships, for example, tracking member participation in meetings and projects, noting attendance of new members and guests at CAC meetings, documenting retention of organizations, and surveying CAC members annually to capture activities, partnerships, and collaborations.
- *Wait for the right time.* Representatives from community organizations and academic researchers have different timelines and availabilities. It is critical to provide adequate time for synergies and relationships to develop.
- *Periodically re-engage community members, faculty, and staff*. Authentic and meaningful CEnR may have starts and stops. For example, the Director of the Center for Addiction Research noted that after meeting with the CAC, the Center’s focus on community outreach and dissemination had decreased; however, Center members wished to continue to build on their initial steps.
- *Continually ensure that the CAC is known as a resource for outreach and dissemination for community members and researchers*. Faculty involved with CAC regularly present to other research organizations within the LHS, as well as meeting with community members and organizations to introduce the CAC and concepts of CEnR, as well as using social media, list serves, and LHS newsletters to make others aware of the CAC.

### The COVID-19 Pandemic

Despite following previous practices of shared leadership and agenda development, engagement and participation in meetings have decreased since they became virtual due to the pandemic. CAC leaders are now discussing how to both increase virtual engagement and resume in-person meetings as warranted.

## Conclusion

CACs provide a tremendous opportunity for developing and nurturing community-academic partnerships and collaborations that solicit and elevate community voices, perspectives, and needs – and in turn, foster authentic CEnR and more comprehensive dissemination of study findings. Developing, sustaining, and harnessing the expertise of community members and organization representatives through a CAC are not easy and take intentionality by all those involved. LHSs can foster these partnerships by building trust and relationships through a shared leadership model, with all partners as equal contributors of their unique knowledge and expertise. Establishing CAC frameworks and processes can maximize the relationship between community organizations and LHSs.

## Footnotes

1 “Stakeholder” is commonly used as an encompassing term for partners, collaborators, and shareholders; however, it groups all parties into one term, despite differential levels of engagement and power. It also has a highly negative connotation for some tribal and indigenous communities (https://www.cdc.gov/healthcommunication/Preferred_Terms.html).

2 The term “Latine” uses a gender-neutral “e,” which replaces the gendered endings “a” and “o” as in “Latina” and “Latino” and is similar to “Latinx.” This term is increasingly used within Latine communities.
